# Co-circulation of the two influenza B lineages during 13 consecutive influenza surveillance seasons in Italy, 2004–2017

**DOI:** 10.1186/s12879-019-4621-z

**Published:** 2019-11-21

**Authors:** Simona Puzelli, Angela Di Martino, Marzia Facchini, Concetta Fabiani, Laura Calzoletti, Giuseppina Di Mario, Annapina Palmieri, Paola Affanni, Barbara Camilloni, Maria Chironna, Pierlanfranco D’Agaro, Simone Giannecchini, Elena Pariani, Caterina Serra, Caterina Rizzo, Antonino Bella, Isabella Donatelli, Maria Rita Castrucci, the Italian Influenza Laboratory Network, Filippo Ansaldi, Rosaria Arvia, Alberta Azzi, Patrizia Bagnarelli, Fausto Baldanti, Maria Rosaria Capobianchi, Silvana Castaldi, Maria Eugenia Colucci, Cristina Galli, Valeria Ghisetti, Andrea Orsi, Elisabetta Pagani, Giorgio Palù, Maurizio Sanguinetti, Riccardo Smeraglia, Fabio Tramuto, Francesco Vitale

**Affiliations:** 1 Department of Infectious Diseases, Istituto Superiore di Sanità (ISS), Viale Regina Elena 299, Rome, Italy; 2 University of Parma, Parma, Italy; 3 University of Perugia, Perugia, Italy; 4 Department of Biomedical Science and Human Oncology, University of Bari, Bari, Italy; 5 University of Trieste, Trieste, Italy; 6 University of Florence, Florence, Italy; 7 Department of Biomedical Sciences for Health, University of Milan, Milan, Italy; 8 University of Sassari, Sassari, Italy; 9 University of Genoa, Genoa, Italy; 10 Department of Biomedical Sciences and Public Health, Virology Lab, Università Politecnica delle Marche, Ancona, Italy; 11 IRCCS Policlinico San Matteo Foundation, Pavia, Italy; 12 INMI “Lazzaro Spallanzani”, Rome, Italy; 13 Amedeo di Savoia Hospital, Turin, Italy; 14 Azienda Sanitaria dell’Alto Adige, Bolzano, Italy; 15 University of Padua, Padua, Italy; 16 Catholic University of Rome, Rome, Italy; 17 Azienda Ospedaliera Dei colli Monaldi-Cotugno-CTO, Naples, Italy; 18 University of Palermo, Palermo, Italy

**Keywords:** Influenza virological surveillance, Influenza B virus, Victoria lineage, Yamagata lineage, Vaccine match, Italy

## Abstract

**Background:**

Since 1985, two antigenically distinct lineages of influenza B viruses (Victoria-like and Yamagata-like) have circulated globally. Trivalent seasonal influenza vaccines contain two circulating influenza A strains but a single B strain and thus provide limited immunity against circulating B strains of the lineage not included in the vaccine. In this study, we describe the characteristics of influenza B viruses that caused respiratory illness in the population in Italy over 13 consecutive seasons of virological surveillance, and the match between the predominant influenza B lineage and the vaccine B lineage, in each season.

**Methods:**

From 2004 to 2017, 26,886 laboratory-confirmed influenza cases were registered in Italy, of which 18.7% were type B. Among them, the lineage of 2465 strains (49%) was retrieved or characterized in this study by a real-time RT-PCR assay and/or sequencing of the hemagglutinin (HA) gene.

**Results:**

Co-circulation of both B lineages was observed each season, although in different proportions every year. Overall, viruses of B/Victoria and B/Yamagata lineages caused 53.3 and 46.7% of influenza B infections, respectively. A higher proportion of infections with both lineages was detected in children, and there was a declining frequency of B/Victoria detections with age. A mismatch between the vaccine and the predominant influenza B lineage occurred in eight out of thirteen influenza seasons under study. Considering the seasons when B accounted for > 20% of all laboratory-confirmed influenza cases, a mismatch was observed in four out of six seasons. Phylogenetic analysis of the HA1 domain confirmed the co-circulation of both lineages and revealed a mixed circulation of distinct evolutionary viral variants, with different levels of match to the vaccine strains.

**Conclusions:**

This study contributes to the understanding of the circulation of influenza B viruses in Italy. We found a continuous co-circulation of both B lineages in the period 2004–2017, and determined that children were particularly vulnerable to Victoria-lineage influenza B virus infections. An influenza B lineage mismatch with the trivalent vaccine occurred in about two-thirds of cases.

## Background

Influenza A(H1N1), A(H3N2) and influenza B viruses are responsible for a significant disease burden during seasonal epidemics in humans [1]. Worldwide, these annual epidemics are estimated to cause about 3–5 million cases of severe illness and 290,000–650,000 deaths [2]. In the past, influenza B viruses were believed to cause milder illness than influenza A, but several studies have recently demonstrated that infections with influenza A and B are clinically indistinguishable and can cause severe complications in both children and adults [1, 3–7]. Originally, influenza B viruses represented a homogenous group but, since the late 1980s, they have evolved into two antigenically and genetically distinct lineages, defined by the reference strains B/Yamagata/16/88 (Yamagata lineage) and B/Victoria/2/87 (Victoria lineage) [8]. In the 1990s, Yamagata-like viruses became predominant worldwide, whereas the Victoria lineage was mainly restricted to East Asia. However, during the 2000/2001 and 2001/2002 seasons, the Victoria-lineage viruses re-emerged in North America and Europe and spread globally [9, 10]. Since then, the two B lineages have been co-circulating worldwide, with variability in terms of geographic distribution and genomic evolution [10–12].

Vaccination is the most effective way to prevent influenza virus infection and its complications. However, due to the constant evolution of influenza viruses, seasonal vaccines are regularly reformulated through continuous global monitoring of the influenza viruses circulating in humans, carried out by the National Influenza Centers (NICs) and World Health Organization Collaborating Centers (WHO-CC) within the WHO Global Influenza Surveillance and Response System (GISRS). Currently, the majority of influenza vaccines used worldwide are trivalent formulations containing one representative A(H1N1) virus, one A(H3N2) virus and only one B strain (B/Victoria or B/Yamagata). The concurrent circulation of the two B lineages over a lengthy period and the limited or absent cross-reactive protection between them have proven particularly challenging in terms of effectiveness of seasonal trivalent influenza vaccines (TIVs), as demonstrated by the frequent lineage-level mismatches occurring in the past between the predominant circulating B viruses and the WHO-recommended B vaccine strain [5, 13–16]. For this reason, quadrivalent influenza vaccines, containing viruses of both B lineages, have recently been introduced in order to reduce the risk of occurrence of B lineage vaccine mismatches [17, 18].

In this retrospective analysis, we provide an update on the circulation of influenza B viruses over a 13-year period (2004–2017) of surveillance in Italy, building upon our previous reports and those at regional level [19–23]. In particular, we document the molecular characteristics of the hemagglutinin (HA) gene in the circulating B strains over the 13 consecutive epidemic seasons and the match between the dominant circulating B-lineage and the lineage included in the TIVs in each season.

## Methods

### Influenza surveillance in Italy and retrospective data collection

Influenza surveillance in Italy is based on a sentinel network of physicians (InfluNet) who report the number of patients with an influenza-like illness (ILI) on a weekly basis and collect respiratory specimens from November to April (i.e. from week 46 to week 17 of the following year), for virological analyses. Each year, a variable proportion of clinical specimens from non-sentinel sources and hospitalized patients are also collected. Virological surveillance is carried out by the NIC at the National Institute of Health (Istituto Superiore di Sanità), in collaboration with the regional influenza laboratory network (InfluNet). Preliminary analyses are performed on clinical samples at regional level and a representative subset of influenza virus-positive samples and/or virus isolates is sent to the NIC and subsequently shared with the WHO-CC for further antigenic and genetic analyses.

For this study, we analyzed all available virological data on laboratory-confirmed influenza B clinical samples collected within the framework of the InfluNet surveillance system, from 2004/2005 to the 2016/2017 season. To increase the number of specimens available for testing, seven regional laboratories of the InfluNet network (i.e. University laboratories of Milan, Trieste, Parma, Perugia, Sassari, Florence and Bari) were involved in the study and collaborated in the characterization analyses, with particular regard to those samples collected during the eight consecutive post-pandemic influenza seasons (i.e. from 2009 to 2017). In accordance with applicable laws and regulations, no clearance from an Ethics Committee is required in Italy for the retrospective analysis of anonymized data collected within the routine influenza surveillance scheme.

### Laboratory testing and characterization analyses

Molecular characterization of the available clinical specimens positive for influenza B virus and/or virus isolates obtained in MDCK cells was performed following RNA extraction, using the QIAamp Viral RNA extraction kit (Qiagen, Hilden, Germany). A real-time RT-PCR assay, recommended by the WHO, was used to discriminate between influenza B lineages [24].

A subset of influenza B viruses (*n* = 491) underwent genetic analysis using conventional Sanger sequencing for the HA1 viral gene. Briefly, viral RNA was transcribed to cDNA using random primers and the HA gene was amplified with segment-specific primers for influenza B [25]. The PCR products were purified using a PCR purification kit (Qiagen, Hilden, Germany) and sequenced using an ABI 3500 genetic analyzer (Applied Biosystems, USA). Sequence alignment was performed using the ClustalW method implemented in the BioEdit program (v. 7.2.5). The phylogenetic trees were constructed using the Neighbor-Joining method and the Kimura 2-parameter model, using MEGA software (v. 6.0). The HA sequences obtained in this study and used in the phylogenetic analysis were entered in the EpiFlu database of the Global Initiative on Sharing All Influenza Data (GISAID) (see Additional file 1: Table S1).

### Data analysis

For each season, we reported the number of samples from ILI patients that were tested, the percentage of influenza positivity, and the number and percentage of influenza-positive cases by viral type (A or B). Data on the circulation of the two B lineages in the country were obtained directly from this study or retrieved from the seasonal influenza database. A lineage-level mismatch between the WHO-recommended B component of the TIVs for use in the northern hemisphere and the circulating B viruses was defined as the percentage of influenza B viruses belonging to a lineage different from that included in the vaccine, for each season in Italy. A complete mismatch was defined for the season when > 60% of circulating B viruses belonged to the lineage not included in the TIV for that season.

For the age distribution and clinical presentation of patients with confirmed B/Victoria and B/Yamagata infections, median values and percentages were compared using Mann-Whitney U tests and Chi-square tests, respectively. The crude and adjusted relative risk (RR) were estimated using the univariable and multivariable log-binomial regression model. Analyses were performed using STATA software v. 11.2 (Stata Corporation, College Station, Texas, US).

## Results

### Circulating patterns of influenza A and B viruses in Italy

**Fig. 1:**
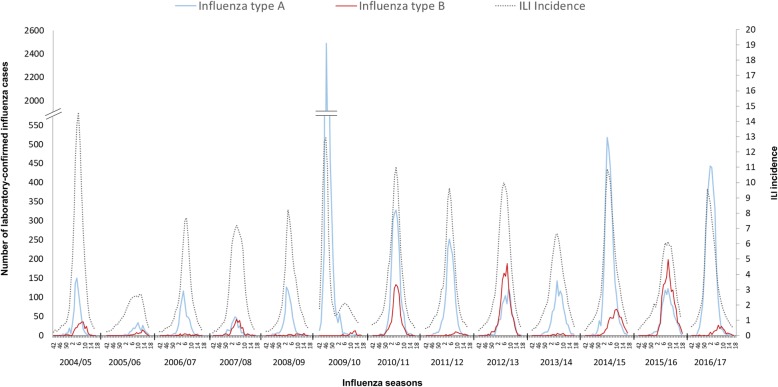
Number of laboratory-confirmed influenza type A and B infections by season in Italy, during the study period (2004/2005–2016/2017). Curves of the Influenza-like illness (ILI) incidence rate (× 1000 population served) are also shown for each epidemic season, by week

During the 13-year study period, a co-circulation of both influenza A and B viruses was observed in Italy each year, although in variable proportions and with a general predominance of type A over type B viruses (Fig. 1, Table 1). Overall, type B viruses circulated on average four weeks later than type A viruses, during winter months, with the exception of three seasons (2010/2011, 2012/2013 and 2015/2016), when the epidemic peak of both influenza A and B almost overlapped (Fig. 1). Across all seasons, 83,479 respiratory specimens were collected and 26,886 (32.2%) of those samples tested positive for influenza viruses. Among these, 81.3% were type A viruses and 18.7% type B viruses, with those percentage values being revised to 76.7 and 23.3%, respectively, when adjusted for differences in total ILI incidence by season (Table 1). The number of influenza-positive samples per season ranged between 307 in 2005/2006 and 3734 in 2014/2015. During the 2009/2010 pandemic season, 6282 samples tested positive for influenza, with the vast majority (> 99%) being A(H1N1)pdm09. Estimated numbers of ILI cases were also reported for each season in Italy (Table 1).

**Table 1:** Number of samples tested and percentages attributable to influenza A and B virus types in Italy, as reported to the virological surveillance system, by season (from 2004/2005 to 2016/2017)

Season	Samples tested	Influenza virus detections (%)	N. of Influenza A viruses (%)	N. of Influenza B viruses (%)	Estimated ILI cases
2004/2005	3226	971 (30)	718 (74)	253 (26)	6336.000
2005/2006	2719	307 (11)	228 (74)	79 (26)	2347.000
2006/2007	1869	643 (34)	607 (94)	36 (6)	3675.000
2007/2008	1870	548 (29)	286 (52)	262 (48)	4692.000
2008/2009	2213	733 (33)	688 (94)	45 (6)	4105.000
2009/2010	16,433	6282 (38)	6230 (> 99)	52 (< 1)	5507.000
2010/2011	9193	2874 (31)	2082 (72)	792 (28)	5917.000
2011/2012	4677	1672 (36)	1610 (96)	62 (4)	5000.000
2012/2013	5535	2114 (38)	894 (42)	1220 (58)	6181.000
2013/2014	4426	1033 (23)	999 (97)	34 (3)	4542.000
2014/2015	10,299	3734 (36)	3133 (84)	601 (16)	6299.000
2015/2016	8985	2457 (27)	1044 (42)	1413 (58)	4877.000
2016/2017	12,034	3518 (29)	3339 (95)	179 (5)	5440.000
All seasons	83,479	26,886 (32.2)	21,858 (81.3)*	5028 (18.7)*	64,918.000

Influenza-like illness (ILI). Source: Influenza Sentinel Surveillance network (InfluNet);

*76.7 and 23.3%, when adjusted for differences in total ILI incidence by season

### Influenza B virus epidemiology and lineage characterization

Influenza B virus detections predominated over type A in only two seasons (2012/2013 and 2015/2016, B = 58%), and similar proportions of B viruses were detected in the 2007/2008 season (B = 48%). Over the entire study period, influenza B accounted for at least 20% of all laboratory-confirmed influenza cases in six seasons (Table 1). Approximately 59% (*n* = 2962/5028) of all laboratory-confirmed influenza B cases were from sentinel sources (community-based infections), whereas the remaining 35 and 6% were from hospitalized patients or other non-sentinel sources, respectively (data not shown).

**Fig. 2:**
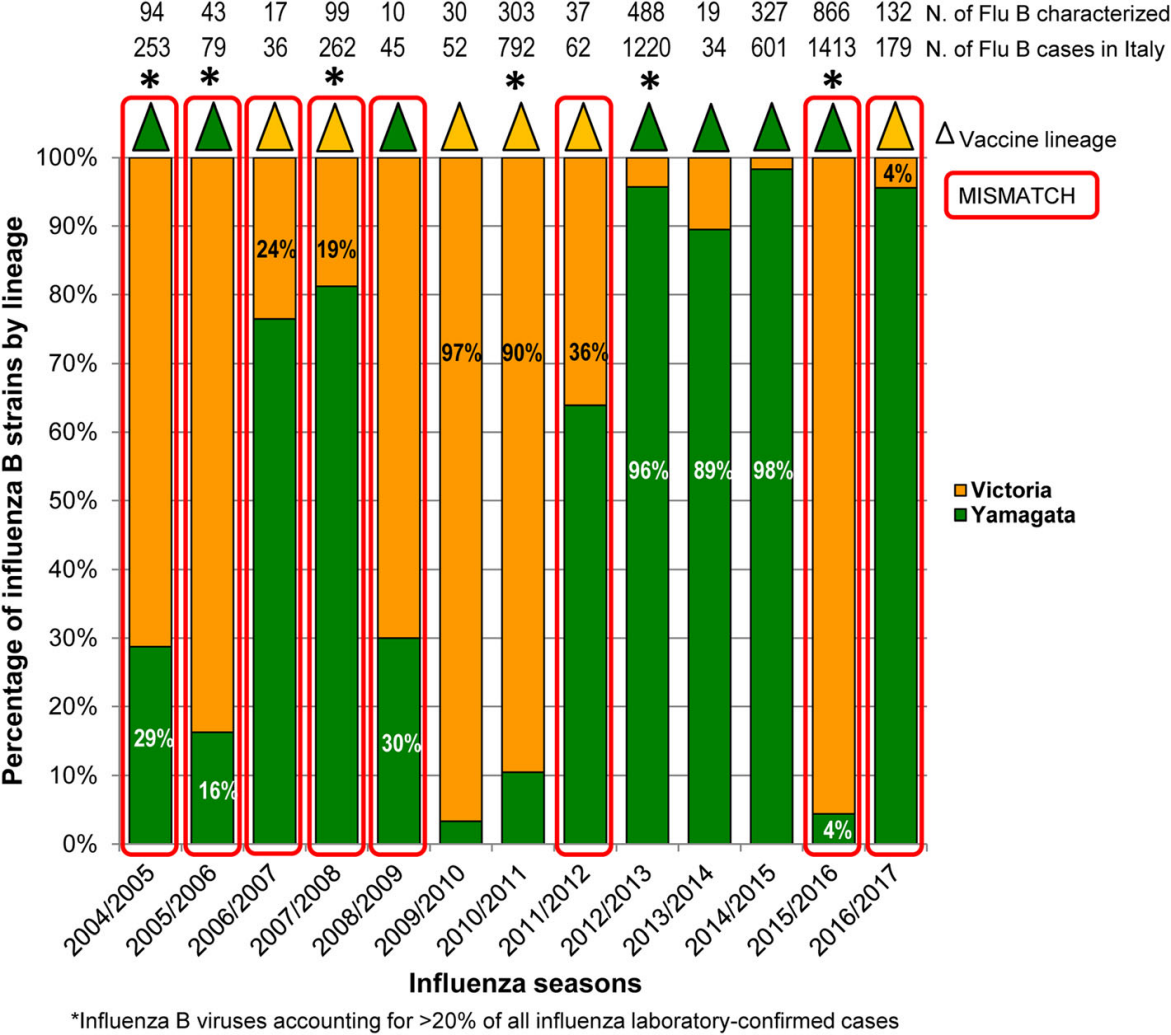
Proportion of B/Victoria and B/Yamagata lineages among characterized influenza B viruses in Italy, by season (2004/2005–2016/2017). The different color of the triangles (B/Victoria in orange; B/Yamagata in green) at the top of each bar represents the recommended influenza B vaccine lineage. The red rectangle indicates a mismatch between the vaccine and the predominant influenza B lineage. Asterisks on the top of the bars indicate the six seasons with a higher circulation of B viruses (> 20% of all laboratory-confirmed cases). The total number of influenza B cases registered in Italy (lower line), along with the number of characterized influenza B viruses (upper line), for each season, are also shown on the top of the graph

In this study, the lineage was characterized or retrieved for 2465 (49%) out of 5028 laboratory-confirmed influenza B infections (Table 2). Of these, B/Victoria and B/Yamagata accounted for 53.3 and 46.7%, respectively. In general, the co-circulation of the two lineages was observed in all seasons considered in Italy, although in different proportions and with alternating patterns, typically every two to three years. Nevertheless, an abrupt annual switch in the predominant lineage occurred in the most recent seasons included in the study, namely 2015/2016 and 2016/2017 (Fig. 2). In nine seasons, one lineage predominated over the other, being identified in > 80% of circulating B viruses, whereas a mixed circulation was observed in the remaining four seasons (2004/2005, 2006/2007, 2008/2009, 2011/2012). During the six seasons with a higher circulation of B viruses (> 20% of all laboratory-confirmed cases), B/Victoria was the lineage most frequently detected in 2004/2005, 2005/2006, 2010/2011 and 2015/2016, whereas B/Yamagata lineage dominated in 2007/2008 and 2012/2013 (Fig. 2, Table 2).

**Table 2:** Proportion of infections caused by lineage-level mismatched influenza B viruses, compared with the vaccine strain, by season (2004/2005–2016/2017) in Italy

Season	Northern Hemisphere TIV B lineage (strain)	N. of B viruses characterized (%)	Prevalent B lineage in Italy (virus variant)	N. of lineage-level mismatched viruses in Italy (%)	Seasonal match (m)/ Mismatch (M)
2004/2005*	Yam (Sha/361/02)	94 (37)	Vic (Ill/13/05)	67 (71)	M
2005/2006*	Yam (Sha/361/02)	43 (54)	Vic (Mal/2506/04)	36 (84)	M
2006/2007	Vic (Mal/2506/04)	17 (47)	Yam (Flo/4/06)	13 (76)	M
2007/2008*	Vic (Mal/2506/04)	99 (38)	Yam (Ban/3333/07)	80 (81)	M
2008/2009	Yam (Flo/4/06)	10 (22)	Vic (Bri/60/08 + Mal/2506/04)	7 (70)	M
2009/2010	Vic (Bri/60/08)	30 (58)	Vic (Bri/60/08)	1 (3)	m
2010/2011*	Vic (Bri/60/08)	303 (38)	Vic (Bri/60/08)	31 (10)	m
2011/2012	Vic (Bri/60/08)	37 (59)	Yam (Wis/01/10)	24 (65)	M
2012/2013*	Yam (Wis/01/10)	488 (40)	Yam (Mas/02/12)	21 (4)	m
2013/2014	Yam (Mas/02/12)	19 (56)	Yam (Phu/3073/13)	2 (11)	m
2014/2015	Yam (Mas/02/12)	327 (54)	Yam (Phu/3073/13)	6 (2)	m
2015/2016*	Yam (Phu/3073/13)	866 (61)	Vic (Bri/60/08)	833 (96)	M
2016/2017	Vic (Bri/60/08)	132 (74)	Yam (Phu/3073/13)	126 (96)	M
All seasons		2465 (49)		1247 (50.6)	

Legenda: Yam: Yamagata; Vic: Victoria; Sha/361/02: B/Shanghai/361/2002; Mal/2506/04: B/Malaysia/2506/2004; Ill/13/05: B/Illinois/13/2005; Flo/4/06: B/Florida/4/2006; Bri/60/08: B/Brisbane/60/2008; Wis/01/10: B/Wisconsin/01/2010; Mas/02/12: B/Massachusetts/02/2012; Phu/3073/13: B/Phuket/3073/2013

* Seasons with at least 20% of all influenza cases due to influenza type B viruses; Mismatch (M): > 60% of the characterized B viruses belonging to the lineage not included in the seasonal TIV

Comparison between the B lineage included in TIVs and circulating B lineages in Italy in the seasons showed that more than 50% (*n* = 1247/2465) of influenza B viruses belonged to the lineage not included in the seasonal TIV (Table 2). A good match (89–98%) was observed only in five seasons, whereas a complete mismatch between the predominant influenza B lineage and vaccine B lineage occurred in the other eight seasons and, in particular, in four out of six seasons with intense influenza B activity (2004/2005, 2005/2006, 2007/2008 and 2015/2016) (Fig. 2, Table 2).

Finally, laboratory-confirmed influenza B cases for which age information for the patient was available were stratified into five different age groups (Table 3). On the whole, the groups most affected by influenza B were children aged ≤14 years (64.5%) whereas the lowest proportion was observed among subjects ≥64 years of age (7.7%). When comparing the two lineages, patients infected by B/Victoria viruses were significantly younger than patients infected by B/Yamagata viruses (median age: 7 years vs. 13 years; *p* < 0.001), with increased detection rates also observed in adults (see Additional file 2: Fig. S1). In particular, the probability of infection with B/Victoria viruses was twice as high in pediatric age groups (0–4 years and 5–14 years) than in the elderly (RR = 2.13 and RR = 2.27, respectively), and slightly higher in hospitalized patients than in community-reported cases (RR = 1.24) (Table 3).

**Table 3:** Proportion of influenza B infections by age group and healthcare setting (Victoria vs Yamagata lineages)*, Italy 2004/2005–2016/2017

Variables	B/Victoria (*N* = 1267)	B/Yamagata (*N* = 1097)	RR_crude_	(95% CI)	RR_adj_	(95% CI)	*p*-value**
	*n*. (%)	*n*. (%)					
Median age (IQR)	7 (9)	13 (41)					< 0.001***
*Age group (years)*							
0–4	302 (23.8)	190 (17.3)	1.91	(1.53–2.39)	2.13	(1.70–2.67)	< 0.001
5–14	655 (51.7)	380 (34.7)	1.97	(1.59–2.45)	2.27	(1.82–2.84)	< 0.001
15–44	198 (15.7)	214 (19.5)	1.50	(1.19–1.90)	1.67	(1.31–2.12)	< 0.001
45–64	54 (4.2)	190 (17.3)	0.69	(0.50–0.95)	0.72	(0.52–0.99)	0.047
> 64	58 (4.6)	123 (11.2)	*Ref*	–	*Ref*	–	–
*Healthcare settings*							
Community	927 (76.6)	810 (77.8)	*Ref*	–	*Ref*	–	–
Hospitals	283 (23.4)	231 (22.2)	1.03	(0.94–1.13)	1.24	(1.15–1.35)	< 0.001

*Crude and adjusted (adj) relative risk (RR) estimated using the univariable and multivariable log-binomial regression model with age bracket of > 64 as a reference class;

*CI: confidence interval*

**p-value of the multivariable binomial negative model;

***p-value of the U Mann-Whitney test; p-values of < 0.05 were considered statistically significant

### Molecular and phylogenetic analysis of influenza B strains

**Fig. 3:**
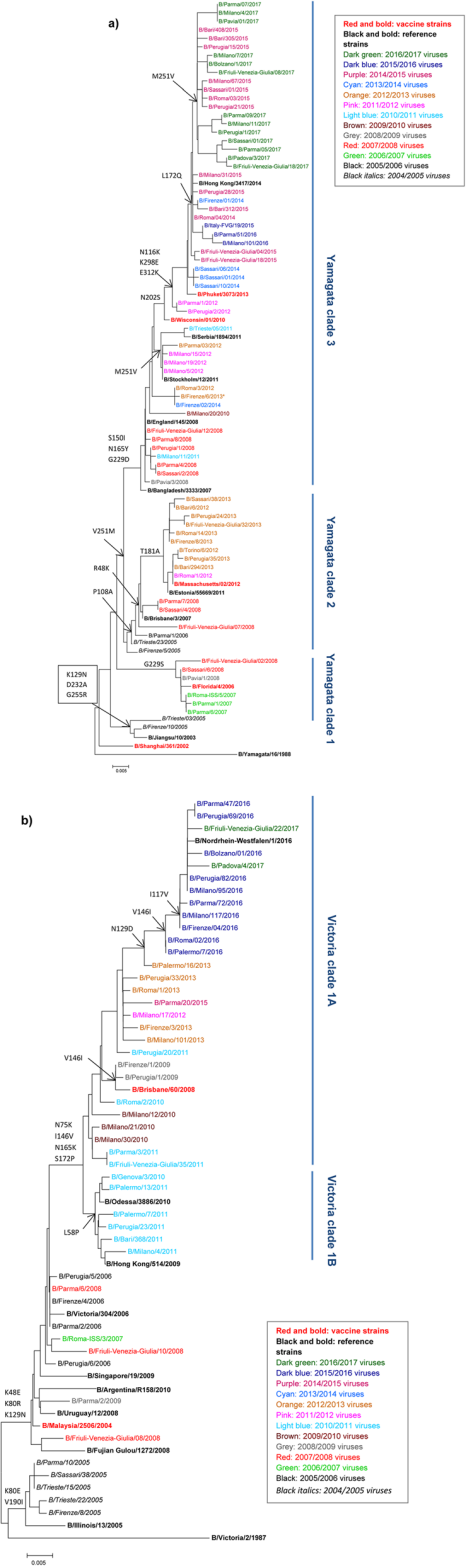
Phylogenetic analysis of the HA1 nucleotide sequences from influenza B/Yamagata (**a**) and B/Victoria (**b**) lineage viruses detected in Italy between 2004 and 2017. Seventy-five HA1 sequences of B/Yamagata (**a**) and 48 of B/Victoria (**b**) viruses were compared with those from the WHO-recommended vaccine strains for the northern hemisphere (in red, bold font) and other reference strains previously reported by WHO-CC (in black, bold font). The strains identified in Italy are colored according to their respective season, as illustrated in the legend. The trees were constructed using the Neighbor-joining method, with Kimura-2 parameter-corrected distances. Amino acid substitutions defining specific genetic clusters are indicated at nodes. The scale bar represents the nucleotide substitutions per site

The HA1 nucleotide sequences of influenza B viruses (*n* = 812, 316 B/Victoria- and 496 B/Yamagata-lineage strains) collected in Italy from 2004 to 2017 were analyzed. Among them, 491 (60.5%) were obtained in this study and the remaining 321 were retrieved from the GISAID and NCBI Influenza Virus Resource databases. Figure 3 shows the phylogenetic relationship of HA1 nucleotide sequences of 123 randomly selected influenza B viruses (see also Additional file 1: Table S1) representing the proportional distribution of different patterns of B/Yamagata (*n* = 75, Fig. 3a) and B/Victoria (*n* = 48, Fig. 3b) in Italy. On average, for each season, the selected HA1 sequences showed more than 99.5% homology with those of other B strains in the same genetic group, not included in the final phylogenetic tree.

The B/Yamagata (Yam) strains were grouped into three main genetic clades with the following reference strains: Yam-1 (B/Florida/4/2006-like strain), Yam-2 (B/Brisbane/3/2007- and B/Massachusetts/02/2012-like strains) and Yam-3 (B/Bangladesh/3333/2007-, B/Wisconsin/01/2010- and B/Phuket/3073/2013-like strains) (Fig. 3a). In 2004/2005, one further small group was detected, represented by the B/Jiangsu/10/2003 reference strain, which included two Italian strains. The remaining B Yamagata strains were characterized by a V251 M substitution, relative to B/Shanghai/361/2002, which was also found in the lone B/Yamagata strain analyzed in the 2005/2006 season. Among the higher proportion of B detections reported during the 2007/2008 season (48%, Table 1), B/Yamagata viruses largely predominated (81%) over those of the B/Victoria lineage (Table 2) and were grouped into the three clades. These were represented by the reference strains B/Florida/4/2006 (Yam-1) (with the G229S change also appearing in viral strains from 2006/2007), B/Brisbane/3/2007 (Yam-2) (with the R48K and P108A changes), and B/Bangladesh/3333/2007 (Yam-3) (with the S150I, N165Y and G229D amino acid changes). From the 2008/2009 to the 2010/2011 season, B/Yamagata viruses circulated again at low levels and were grouped mainly in the Yam-3 clade. In the subsequent 2011–2015 period, B/Yamagata viruses prevailed over B/Victoria viruses and were grouped into the Yam-2 and Yam-3 clades. In particular, the 2012/2013 season was characterized by an intense circulation of B viruses (58%, Table 1), largely belonging to the Yam-2 clade (*n* = 158/174, 91%) related more to the B/Massachusetts/02/2012 reference strain and less to the Yam-3 clade, represented by the B/Wisconsin/1/2010 vaccine strain. For this reason, B/Massachusetts/02/2012 was included in the TIV formulation for the subsequent 2013/2014 and 2014/2015 seasons although those seasons were exclusively dominated by the circulation of Yam-3 viruses, related to B/Phuket/3073/2013, with N116K, K298E and E312K changes compared to B/Wisconsin/1/2010. In 2015–2017, all the B/Yamagata viruses circulating in Italy clustered with the 2015/2016 B/Phuket/3073/2013 vaccine strain, with the additional L172Q and M251 V amino acid changes. No further viruses belonging to the Yam-2 clade were observed in Italy following the 2012/2013 season.

The B/Victoria-like strains identified in Italy were grouped with the following reference viruses that emerged over time: B/Illinois/13/2005, B/Malaysia/2506/2004 and B/Brisbane/60/2008. In particular, the HA genes of the strains circulating in the 2005–2009 period in Italy were closely related to the B/Malaysia/2506/2004 vaccine strain, sharing the K48E, K80R and K129 N amino acid changes. Although circulating in very low proportions over the whole season, most (66%) of the 2008/2009 B/Vic strains fell within a new clade (Vic-1), represented by B/Brisbane/60/2008, with N75K, N165K and S172P changes compared to B/Malaysia/2506/2004. The following six seasons, from 2009/2010 to 2014/2015, were characterized by the circulation of viruses belonging to Vic-1 clade, carrying the additional I146V substitution compared to B/Brisbane/60/2008. Phylogenetic analysis shows that the Vic-1 clade is further divided into 1A and 1B subclades, with Vic-1B viruses (B/Hong Kong/514/2009-like strains) characterized by an L58P amino acid change, as found in more than half (*n* = 44/71; 62%) of the strains analyzed for 2010/2011. All the remaining B/Victoria strains from 2010/2011 onwards clustered differently with the B/Brisbane/60/2008 genetic group (renamed Vic-1A) and those identified between 2015 and 2017 carried two additional amino acid changes (I117V and N129D). The only exception was represented by four B/Victoria strains from the 2015/2016 season that were characterized by a different pattern of mutations (i.e. I97M, K209 N and T258A), which were more closely related to B strains circulating in previous seasons [23].

A comparison was made of the HA1 amino acid sequences of the Italian B viruses collected in the period 2012–2017 (145 Vic-1, 169 Yam-2 and 236 Yam-3) with the respective vaccine strains within the four major epitope domains comprising the 120-loop (position 116–137), 150-loop (141–150), 160-loop (162–167), and the 190-helix (194–202) [26]. Among these, the 120-loop appeared to be the most frequently mutated region in field strains (Table 4) [27]. In particular, three substitutions were detected more frequently within the 120-loop: N116K (shared by 88.6% of Yam-3 viruses), I117V and N129D (shared by 80.7 and 87.6% of Vic-1 viruses, respectively). The I146V substitution was found in the 150-loop region of 13.8% of Vic-1 viruses, and mostly identified in those strains circulating in the 2012/2013 and 2014/2015 seasons. Within the 190-helix, most Vic-1 (97.9%), Yam-2 (100%) and Yam-3 (99.6%) viruses acquired a potential glycosylation site with D197N/D196N substitutions (based on Brisbane/60/2008- or Massachusetts/02/2012- and Wisconsin/01/2010- vaccine strain numbering) [26]. Moreover, the S202 N substitution was identified in 8.9% of Yam-3 viruses, and particularly in those circulating from 2012 to 2015. Finally, additional amino acid substitutions were found within the four antigenic epitopes and only detected in a few circulating field strains (Table 4).

**Table 4:** Amino-acid substitutions found in the HA protein of 550 influenza B viruses (145 Victoria and 405 Yamagata) analyzed in this study and detected in Italy during the period 2012–2017

Subunit	Residue at site*	Vic-1 (*N* = 145)	Yam-2 (*N* = 169)	Yam-3 (*N* = 236)
HA1	120-loop (116–137)	I117V (117)	R118K (1)	N116K (209)
		V124A (1)	S120 T (7)	N116R (5)
		N129D (127)	T121S (10)	Q122K (9)
			N123K (3)	N123T (4)
			D126N (3)	D126N (1)
	150-loop (141–150)	I146V (20)	G141R (1)	S148 N (1)
		T147A (1)	S150I (1)	I150V (1)
	160-loop (162–167)		N165Y (1) D163N (1)	D163G (1)
	190-helix (194–202)	D197N (142)	D196N (169)	D196N (235)
		N197D (3)	T198N (1)	D196Y (1)
		T199A (2)	N202S (2)	K197R (4)
		T199I (1)		S202 N (21)
		A202T (1)		

*Amino acid substitutions reported in this table are compared with vaccine strains of respective clades and numbered according to respective vaccine strains (B/Brisbane/60/2008 for Vic-1, B/Massachusetts/02/2012 for Yam-2, B/Wisconsin/01/2010 for Yam-3). The number of influenza viruses carrying the substitution is indicated inside parenthesis

## Discussion

Influenza B viruses represent a significant cause of respiratory infections in humans that generally tend to be overlooked because of the dominance of influenza A [1, 3–5, 28]. In this study, we provide a comprehensive picture of the circulation of the two influenza B lineages in Italy, on the basis of 13 consecutive years of national influenza surveillance data, from 2004/2005 to 2016/2017. Overall, our data show that both influenza type A and B viruses, and each lineage of influenza B, co-circulated annually in varying proportions. Although influenza A viruses predominated in the majority of the seasons considered, type B viruses accounted for 20% or more of all influenza detections in six seasons, playing a significant epidemic role in the population. Moreover, we found that influenza B was responsible for nearly 19% of all laboratory-confirmed influenza cases, a figure thus comparable to the global proportion (20%) reported by Caini et al. during the period 2000–2013 in 26 countries around the world, including Italy [29]. Influenza B virus detections exceeded those of influenza A in only two seasons (2012/2013 and 2015/2016), with a switch in the predominant B lineage. Indeed, a change in the relative predominance of the two influenza B lineages was essentially observed every two/three years, with the exception of the three more recent seasons, when a lineage switch occurred each year, with a sudden shift from B/Yamagata in 2014/2015 to B/Victoria in 2015/2016 and back to B/Yamagata in 2016/2017. During the study period, a good lineage-level match with the WHO-recommended TIV B component was observed in five seasons only, whereas the predominant lineage differed in the remaining eight seasons, causing a complete vaccine mismatch. In those seasons with considerable influenza B activity, a mismatch was observed in four seasons (2004/2005, 2005/2006, 2007/2008 and 2015/2016). These results largely reflect trends observed in other European countries, with the exception of the 2004/2005 season, when the majority of characterized B isolates in Europe belonged to the Yamagata lineage [5, 30]. Overall, our data further highlight the unpredictability of influenza B circulation, which makes it challenging to predict which B lineage will predominate in the upcoming influenza season.

This study did not permit any vaccine effectiveness estimates related to the occurrence of B mismatches because of the limited availability of data on vaccination status. However, the potential clinical impact of a B vaccine mismatch is mainly dependent on the proportion of circulating influenza B viruses, as well as pre-seasonal immunity in the population. Even though our analysis is based on observational data only, we can assume that the overall impact was minor in four out of eight seasons with a complete B mismatch, given the very low proportion of circulating influenza B viruses. For the other seasons, a much higher impact can be expected, as occurred in 2007/2008 when influenza B was found in 48% of the influenza positive samples in Italy and 81% of the characterized B strains were vaccine-mismatched B/Yamagata lineage viruses. Similarly, in 2015/2016, influenza B was found in 58% of the influenza positive samples and 96% of the characterized viruses belonged to the vaccine-mismatched B/Victoria lineage. Notably, mismatched influenza B viruses were also reported in seasons with a lineage-level vaccine match. In particular, a high proportion (91%) of B/Yamagata strains circulating in Italy during the 2012/2013 season were related to B/Massachusetts/02/2012-like (Yam-2 clade) strains, antigenically distinguishable from the B/Wisconsin/1/2010 (Yam-3) vaccine strain. Interestingly, B/Yamagata lineage viruses also predominated in the following two seasons in Italy and worldwide, with a shift to clade 3 viruses, closely related to the B/Phuket/3073/2013 strain and differing from the B/Massachusetts/02/2012 vaccine strain by at least 11 amino acid substitutions in the HA1 [12, 31–33]. Thus, an alternation of two antigenically distinct B/Yamagata clades might have reduced the vaccine’s ability to protect against these viruses, although they belong to the same lineage. Further studies are needed to evaluate the extent of cross-reactivity between influenza B viruses from distinct clades, and even from different lineages, and thus establish the real impact on vaccine effectiveness.

Phylogenetic analysis of influenza B viruses circulating in Italy over the entire study period confirmed the co-circulation of both lineages during each season, and revealed a mixed circulation of distinct evolutionary viral variants with different levels of match to the vaccine strains. In particular, a gradual drift was observed in both lineages and further clades and subclades were identified in each lineage. However, following the 2012/2013 season, B/Yamagata viruses of clade 3 dominated in the influenza B virus population, thereafter showing only limited amino acid variation. Similarly, the B/Victoria lineage segregation into Vic-1A and Vic-1B subclades was not detected further in Italy after the 2010/2011 season and Vic-1A (B/Brisbane/60/2008-like) viruses became dominant, displaying only two important amino acid substitutions in the antigenic 120-loop region of HA, as compared to B/Brisbane/60/2008 [34].

With regard to influenza B virus circulation among the population in Italy, most influenza B infections (> 60%) were found in children aged ≤14 years, with the highest proportion (nearly 44%) among school-age children (5–14 years), in line with previous studies globally [5, 15, 29, 35–37]. In particular, our study confirmed a higher proportion of B/Victoria virus infections in children than those caused by B/Yamagata viruses [38–42]. A similar distribution of B/Victoria and B/Yamagata strains was observed in the community and in hospitals, although there was a slightly higher probability of B/Victoria infections in hospitalized patients. Indeed, previous studies have reported a greater proportion of B/Yamagata virus infections in hospital inpatients across two Italian regions [21]. The different time period and number of patients with these characteristics may account for any difference observed in the outcomes for the two studies. Furthermore, the potential association between B lineage and age of infected patients and the increased virulence of the B/Victoria lineage suggested by others are still controversial and require further investigation [39, 40, 43–45].

There are some limitations in this study. Only laboratory-confirmed influenza cases were included in the study, and this could underestimate the real proportion of circulating viruses and the true incidence of influenza infections in the population. In addition, there were increased notifications received by hospitals in later years, as a result of a much higher demand for testing from the 2009 H1N1 influenza pandemic onwards. Lastly, we were only able to provide B lineage information on almost half of all laboratory-confirmed influenza B notifications. Nevertheless, we can assume that these results would largely reflect the circulation of the two influenza B lineages in Italy.

## Conclusions

This study provides information on the viral evolution and incidence of influenza B virus infections in Italy over a 13-year period. Continuous co-circulation of both B lineages highlights the complexity of antigenic variation in influenza B viruses and the differences in the epidemiological profile of the target population. In addition, the frequent occurrence of B-lineage mismatch with the TIV, as observed during the 2004–2017 seasons, likely contributed to the adoption of quadrivalent vaccines for influenza vaccination programs in the country.

## Supplementary information

**Additional file 1: Table S1.** GISAID accession numbers (AN) for hemagglutinin (HA) of influenza B viruses generated in this study (*) and used for the phylogenetic analysis, along with other Italian sequences retrieved from GISAID or NCBI database and reference and WHO vaccine strains (in bold).

**Additional file 2: Figure S1.** Violin plot comparing the median values (white dots), interquartile range (thick blue bar in the center) and distributions of age between influenza B/Victoria- and B/Yamagata-lineage cases.

## Data Availability

HA sequences of B viruses are available in the GISAID database.

## Abbreviations

cDNA: complementary Deoxyribo-Nucleic Acid
HA: Hemagglutinin
ILI: Influenza-like illness
MDCK cells: Madin-Darby canine kidney cells
NIC: National Influenza Centre
RNA: Ribonucleic acid
RT-PCR: Reverse Transcription-Polymerase Chain Reaction
TIV: Trivalent influenza vaccine
WHO: World Health Organization
WHO-CCs: WHO Collaborating Centres
WHO-GISRS: WHO Global Influenza Surveillance and Response System
