# Supplementary figures and images for: Co-circulation of the two influenza B lineages during 13 consecutive influenza surveillance seasons in Italy, 2004–2017
Source: BMC Infect Dis. 2019 Nov 21;19:990. doi: 10.1186/s12879-019-4621-z (PMC6873537; doi:10.1186/s12879-019-4621-z)

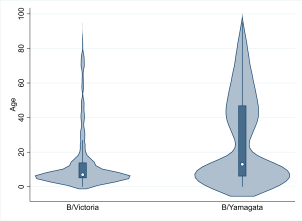

Supplement: Supplementary file 2 — Additional file 2: Figure S1. Violin plot comparing the median values (white dots), interquartile range (thick blue bar in the center) and distributions of age between influenza B/Victoria- and B/Yamagata-lineage cases. [file 12879_2019_4621_MOESM2_ESM.tif]
